# Activation of dynamin-related protein 1 - dependent mitochondria fragmentation and suppression of osteosarcoma by cryptotanshinone

**DOI:** 10.1186/s13046-018-1008-8

**Published:** 2019-01-28

**Authors:** Jia-Hau Yen, Hung Sen Huang, Chia Ju Chuang, Sheng-Teng Huang

**Affiliations:** 1 Research Cancer Center for Traditional Chinese Medicine, Department of Medical Research, China Medical University Hospital, Taichung, Taiwan; 2 Department of Chinese Medicine, China Medical University Hospital, No. 2, Yude Rd, North District, Taichung, 40447 Taiwan; 3 School of Chinese Medicine, China Medical University, Taichung, Taiwan; 4 Chinese Medicine Research Center, China Medical University, Taichung, Taiwan; 5 Research Center for Chinese Herbal Medicine, China Medical University, Taichung, Taiwan; 6 Tainan Municipal An-Nan Hospital, China Medical University, Taichung, Taiwan

**Keywords:** Cryptotanshinone (CPT), Drp1, Mitochondria fragmentation, Osteosarcoma

## Abstract

**Background:**

Discovering how to regulate mitochondrial function to reduce cancer growth holds great potential for future cancer therapy development. Here we explore the effects of cryptotanshinone (CPT), a natural product derived from *Salvia miltiorrhiza*, on mitochondria of osteosarcoma (OS) both in vitro and in vivo, and further elucidate the underlying molecular mechanisms.

**Methods:**

Cytotoxicity in the CPT treated OS cells was analyzed by flow cytometry, CCK8, TUNEL assay and colony formation assays. Flow cytometric analysis was performed to evaluate the effect of CPT on cell cycle of OS cells. Mitochondrial morphology was examined by staining with the mitochondrial membrane potential -sensitive fluorochrome, MitoTracker Red (CMXRos). Immunoblotting, confocal-immunofluorescence staining, co-immunoprecipitation were used to examine the expression and interaction between CPT-mediated Drp1 and Bax. Finally, the synergistic effect of CPT on OS cells was validated using a mouse xenograft tumor model.

**Results:**

In this study, we found CPT treatment induced S-phase arrest, apoptosis, and mitochondrial fragmentation in OS cells. CPT also effectively activated caspase-dependent apoptosis, which could be blocked by pan-caspase inhibitor Z-VAD-FMK. Moreover, we herein provide evidence that treatment with CPT resulted in mitochondrial fragmentation, which is mediated by dynamin-related protein 1 (Drp1), a key mediator of mitochondrial fission. Pursuing this observation, downregulation of Drp1 via silencing RNA could abrogate the induction of apoptosis and mitochondrial fragmentation induced by CPT. Finally, we demonstrate that CPT induced Drp1, which interacted directly with Bcl-2-associated X protein (Bax), which contributed to driving Bax translocation from the cytosol to the mitochondria.

**Conclusions:**

Our findings offer insight into the crosstalk between mitochondrial fragmentation and inhibition of osteosarcoma cell growth in response to CPT.

**Electronic supplementary material:**

The online version of this article (10.1186/s13046-018-1008-8) contains supplementary material, which is available to authorized users.

## Introduction

Osteosarcoma is the most commonly occurring form of malignant bone tumor. Specifically, osteosarcoma is the development of cancer in areas of postnatal bone growth and bone remodeling. Although osteosarcoma can affect people of all ages, it most often occurs in children and teens who are still growing, which indicates genetic and molecular alterations that disrupt osteoblast differentiation are important factors in the etiology of the disease. Tumors occur predominantly around the knee, in either the femur (thighbone) or tibia (shinbone) [1]. However, osteosarcoma can also affect other parts of the body and can even develop outside of bones, in soft tissues (extraskeletal osteosarcoma), especially in elderly patients. Treatments of osteosarcoma include surgery, radiation, chemotherapy, or a combination of radiotherapy and chemotherapy; however, such therapies are often negatively characterized by toxicity and side effects. Additionally, considering the long-term and short-term toxicities of chemotherapeutic agents commonly used to treat osteosarcoma, a more promising approach targets the development of effective, and nontoxic therapeutic strategies, using active constitutive agents extracted from natural sources.

Mitochondria play an important role in the production of energy within cells, and are essential in the regulation of cellular life and death. In most healthy mammalian cells, mitochondria exhibit tubular, reticular, or networked morphology which is regulated by dynamic remodeling via the balance between fusion and fission events [2, 3]. Mammalian large GTPases regulate mitochondrial fusion: mitofusins (Mfn) 1 and 2 mediate outer mitochondrial membrane (OMM) fusion, while optic atrophy 1 (Opa1) is responsible for inner mitochondrial membrane (IMM) fusion. Conversely, dynamin-related protein 1 (Drp1) is the master regulator of mitochondrial division in most eukaryotic organisms [4]. Together, Drp1 with its OMM receptors Fission 1 (Fis1), mitochondrial fission factor (MFF), and mitochondrial elongation factor 1 (Mief1) mediates mitochondrial fission [5–7]. Inhibition of Drp1 by either expression of a Drp1 dominant mutant or RNA interference leads to increased length and interconnectivity of mitochondrial tubules, thereby inhibiting the fission process and preventing cell death [8]. Importantly, excessive mitochondrial fission and mitochondrial structural disarray has been linked to increased mitochondrial production of reactive oxygen species (ROS), impaired function, and activation of cell death [9, 10]. Evidence has emerged indicating that Drp1 and Bax mitochondrial translocation is a crucial step for induction of apoptosis [11–13].

Danshen root, has been used in traditional Chinese medicine to treat coronary heart disease for thousands of years [14, 15]. Cryptotanshinone (CPT), a natural quinoid diterpene isolated from Danshen root, has been reported to exhibit inhibitory effects on STAT3 activation; furthermore, it has demonstrated other pharmacological effects in the treatment of cardiovascular diseases [16], anti-inflammation [17], and neuron protection [18]. Recently, CPT has has been shown to display diverse anticancer properties against many tumors occurring in humans, such as prostate cancer, leukemia, gliomas, lung carcinomas, hepatic carcinomas, pancreatic cancer, breast cancer, colorectal cancer, and melanoma cancer [19–23]. However, the specific effects of CPT on osteosarcoma have yet to be elucidated. The purpose of the present study is to investigate the mitochondrial morphology-function relationship to gain insight into the anticancer effects of CPT.

## Materials and methods

### Mice

All experiments were done under Institutional Animal Care and Use Committee approval at China Medical University (Taichung, Taiwan) (2017–077). NOD/SCID (NOD CB17-Prkdcscid/NcrCrl, male, 5 weeks of age) mice were obtained from BioLASCO Taiwan Co., Ltd. All mice were housed in specific pathogen−free conditions. During the entire maintenance period, all mice were permitted free cage activity without joint immobilization. The initial body weights of the mice were between 20 and 23 g. After subcutaneous injection of 143B osteosarcoma cells into the back of NOD/SCID mice, the mice were treated with or without CPT (10 or 20 mg/kg). CPT was diluted in DMSO: Ethanol: Normal Saline: Hydroxypropyl-beta-cyclodextrin (HP-beta-CD) = 1:3:3:3 and heated to 60 °C before injection to mice. Seven days after 143B osteosarcoma cell injection, IP injection with CPT was carried out every other day followed by sacrifice at day 45 of tumor cell inoculation. The tumors were removed, weighed, and fixed for use in immunohistochemical experiments. All experiments were carried out using 5 mice in each group, with three independent experiments.

### Materials

Cryptotanshinone (C5624), JC-1 Dye (T3168), MitoTracker Red CMXRos (M7512) and Z-VAD-FMK (V116) were purchased from Sigma-Aldrich (USA). The primary antibodies against β-actin (ab151318), α-tubulin (ab4074), Caspase-3 (ab44976), Caspase-8 (ab25901), Caspase-9 (ab184786), Bcl-2 (ab182858), Bax (ab32503), Bid (ab10640), Bad (ab62465), Drp1 (ab56788)), Opa1 (ab119685), Mfn-1 (ab57602), Mfn-2 (ab124773), and Hsp60 (ab46798) were purchased from Abcam (UK). Annexin V apoptosis detection kit (55647) was purchased from BD (USA).

### Cell culture

The human osteosarcoma (OS) cell line 143B and MG63 cells were grown in DMEM supplemented with 10% FBS and 100 units/ml penicillin and 100 μg/ml streptomycin (HyClone, USA) at 37 °C in a 5% CO_2_ incubator. To examine whether CPT could induce cell death in osteosarcoma, cells were treated with different doses of CPT for 24 h.

### Cytotoxicity assay

Cell Counting Kit-8 (CCK-8) was obtained from Dojindo (Dojindo Co. Ltd., Japan). Briefly, cells were plated in 96-well plates at a density of 1 × 10^4^ cells per well and cultured in the growth medium. At the indicated time points, the number of cells in triplicate wells was measured using the absorbance at 450 nm of reduced WST-8 (2-(2-methoxy-4-nitrophenyl)-3-(4-nitrophenyl)-5-(2,4-disulfophenyl)-2H-tetrazolium, monosodium salt).

### TUNEL assay

The terminal deoxynucleotidyl transferase-mediated dUTP nick-end labeling (TUNEL) assay was used to evaluate the apoptotic response of tumor cells with a kit from Roche Applied Science (Germany). The cells grew on coverslips fixed by 4% PFA for 30 min at room temperature and washed 3 times by PBS and then incubated with 0.1% Triton X-100 for 2 min and washed by PBS. TUNEL assay was performed according to the manufacturer’s instruction (Roche Applied Science, Germany). After washing in PBS, nick-end labeling was visualized by immersing sections in DAB solution with 3% hydrogen peroxide and counterstained with methyl green. Finally, the sections were counterstained with Mayer’s hematoxylin, washed in water, and mounted. All slides were observed under light microscopy.

### Histological analysis

The histological observation was performed by staining with hematoxylin/eosin (H&E), Giemsa, and trichrome stains using paraffin sections. For H&E staining, paraffin-embedded sample slides were de-paraffinized, hydrated, and then stained with hematoxylin for 1 min. After rinse, the slides were stained with eosin for 5 min, rinsed, and sealed with cover slips. Tissue sections from tumor mass, kidneys, spleen, liver, heart, and lungs were used. The slides were counterstained with hematoxylin and mounted. To determine the effect of CPT on expression of Ki67, PCNA, Caspase-3, Caspase-8, Caspase-9, Bcl-2, Bax, Bid, Bad, Drp1, Opa1, Mfn1, and Mfn2 by immunohistochemistry, the slides were blocked in 5% bovine serum for 15 min, followed by incubation with the primary antibody at 4 °C overnight in a moist chamber. The sections were then incubated with the corresponding secondary antibodies. The antigen-antibody complex was detected by Dako Liquid DAB + Substrate-Chromogen System (Dako, Carpinteria, CA). All slides were examined under light microscopy. Giemsa stain and trichrome stain were performed according to the manufacturer’s instruction of Giemsa Stain Kit (ab150670) and Trichrome Stain Kit (ab150686) from Abcam (UK). All slides were examined under light microscopy.

### Short interfering RNA (siRNA) transfection

Non-targeting siRNA and siRNAs targeting human Drp1 (H00010059-R01) were obtained from Abnova Corporation (Taipei City, Taiwan) (Additional file 1: Table S1). The siRNAs were transfected into the cells using siRNA transfection reagent (Santa Cruz Biotechnology; cat. no. sc-29,528) according to the manufacturer’s protocol. After overnight incubation, cells were treated with or without CPT for 24 h.

### Flow cytometry

Cells were placed in 6-well plates and treated with different combinations for 24 h. The cells were harvested, washed twice with cold PBS, and stained with FITC-conjugated annexin V and propidium iodide for 15 min (BD, USA) in the dark, or stained with 10 μg/mL JC-1 (Thermo Fisher Scientific, MA, USA) in DMEM medium at 37 °C for 30 min. For cell cycle analysis, the cells were then fixed with cold 70% ethanol overnight at − 20 °C, and propidium iodide staining was performed. The stained cells were assessed by flow cytometry (BD FACSLyric, USA), and analyzed by FlowJo V10 software.

### Western blot analysis

The tumor masses or CPT-treated cells were harvested and total cell protein was extracted using whole cell lysis buffer. The protein concentrations were determined by the Bradford method (Bio-Rad, CA, USA). Samples with equal amount of protein were subjected to 8–15% sodium dodecyl sulfate polyacrylamide gel electrophoresis (SDS-PAGE) and transferred onto a polyvinylidenedifluoride (PVDF) (Millipore, Bedford, MA, USA) membrane. The membrane was incubated at room temperature in blocking solution (5% nonfat milk) for 1 h followed by incubation for 2 h in blocking solution containing an appropriate dilution of anti- Caspase-3, Caspase-8, Caspase-9, Bcl-2, Bax, Bid, Bad, Drp1, Opa1, Mfn1, Mfn2 antibody (Abcam, UK). After washing, blots were then probed with appropriate secondary horseradish peroxidase (HRP)-conjugated secondary antibodies (Jackson ImmunoResearch, West Grove, PA) and detected by an ECL detection system (Millipore) and scanned by MultiGel-21 (Top Bio, Taiwan). β-actin served as internal control. Cytosolic and mitochondrial protein extractions were performed according to manufacturer’s protocol from Thermo Scientific (Waltham, MA, USA). HSP60 and α-tubulin were used as mitochondrial and cytosolic markers, respectively.

### Confocal laser microscopy

In order to measure transitions in the mitochondrial morphology, the CPT-treated cells were reacted with 10 nM MitoTracker Red CMXRos probe (Invitrogen Corp., Carlsbad, CA, USA) for 20 min at 37 °C, according to the manufacturer’s instructions. After being washed twice in cold PBS, the live cells were visualized under a Leica confocal laser scanning microscope (TCS SP8, Wetzlar, Germany). MitoTracker Red was monitored at an excitation wavelength of 579 nm to locate mitochondria. Fragmented mitochondria were shortened, punctate, and sometimes spherical, whereas filamentous mitochondria showed a long thread-like tubular structure [24].

### Statistical analysis

All experiments were performed in triplicate and data presented in a representation of three individual experiments. Statistical analyses were performed using GraphPad Prism statistical software (version 6, GraphPad Software, Inc., San Diego, CA). Data was represented as means ± standard deviation (SD) of three independent experiments. One-way ANOVA was carried out when multiple comparisons were evaluated. Values were considered to be significant at *p* less than 0.05.

## Results

### CPT induced OS cell death and cell cycle arrest

**Fig. 1:**
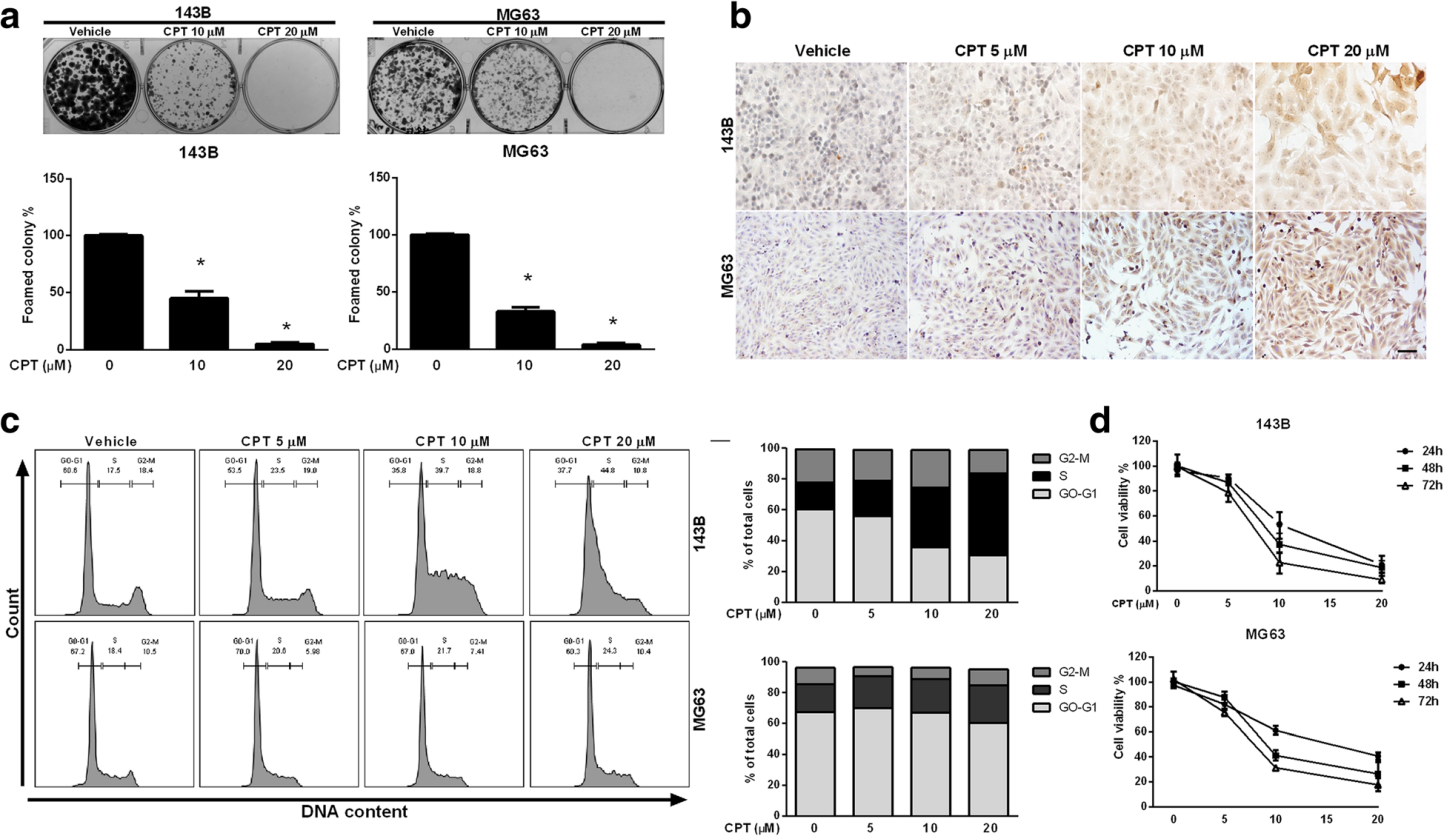
CPT induces S phase arrest and cells death in human OS cells. **a** Clonogenicity of OS cells treated with various concentrations of CPT (as indicated). **b** Representative images of TUNEL staining in OS cells treated with various concentrations of CPT (as indicated). Bar represents 50 μm. **c** OS cells were treated with control and CPT (as indicated) for 24 h. Flow-cytometric analysis and quantification of distribution of cell cycle were assessed. **d** OS cell viability following treatment with the various concentrations of CPT for 24, 48, and 72 h. CCK-8 assay was used to assess OS cell proliferation. The results were expressed as the means ± SD from three independent experiments. *P < 0.05, significantly different compared with control

The properties of growth inhibition and induction of apoptosis by CPT have previously been reported in renal cell carcinoma and colorectal cancer cell lines [25]. In the present study, we performed a colony formation assay to analyze the effect of CPT on clonogenic survival of 143B and MG63 osteosarcoma cell lines. After treatment with different concentrations of CPT (10 and 20 μM) for 3 weeks, dose-dependent and statistically significant inhibition of cell colony formation was observed in the presence of CPT (Fig. 1a). To further clarify whether CPT induces cancer cell apoptosis, we detected apoptosis by TUNEL assay. Compared with the control group, the apoptotic rates and TUNEL positive cells in the CPT-treated groups were increased in both 143B and MG63 cells (Fig. 1b). To further investigate the potential mechanism via which CPT repressed 143B and MG63 cell growth, cell cycle analysis was also performed after CPT treatment for 24 h. As shown in Fig. 1c, CPT induced obvious S-phase arrest at concentrations of 10 and 20 μM, while vehicle control did not. To determine the inhibitory effects and cytotoxicity of CPT in OS cells, 143B and MG63 cells were treated with various concentrations of CPT for 24, 48, and 72 h, and subsequently assayed by Cell Counting Kit-8 (CCK-8) (Fig. 1d). The IC50 values were 10.99 μM (24 h), 8.9 μM (48 h), and 7.2 μM (72 h) for 143B cells, while the IC50 values for MG63 were 14.7 μM (24 h), 9.9 μM (48 h), and 7.7 μM (72 h). We further examined the cell viability of normal cell lines including mouse mesenchymal stem cell (MMSC), human mammary epithelial cell (H184) and human keratinocyte cell line (HaCaT) to indicate cytotoxic effect induced by CPT. Our results demonstrated that CPT had no cytotoxicity with various concentrations for 24 and 48 h treatments (Additional file 2: Figure S1). Furthermore, cell cycle-regulating molecular machinery were measured by western blotting, the protein levels of Cyclin A and Cdk2 were increased, but Cyclin D1 was decreased with dose dependent manner of both OS cells (Additional file 3: Figure S2)which indicated the S-phase arrest induced by CPT treatment.

### CPT treatment inhibited osteosarcoma progression in NOD-SCID mice

**Fig. 2:**
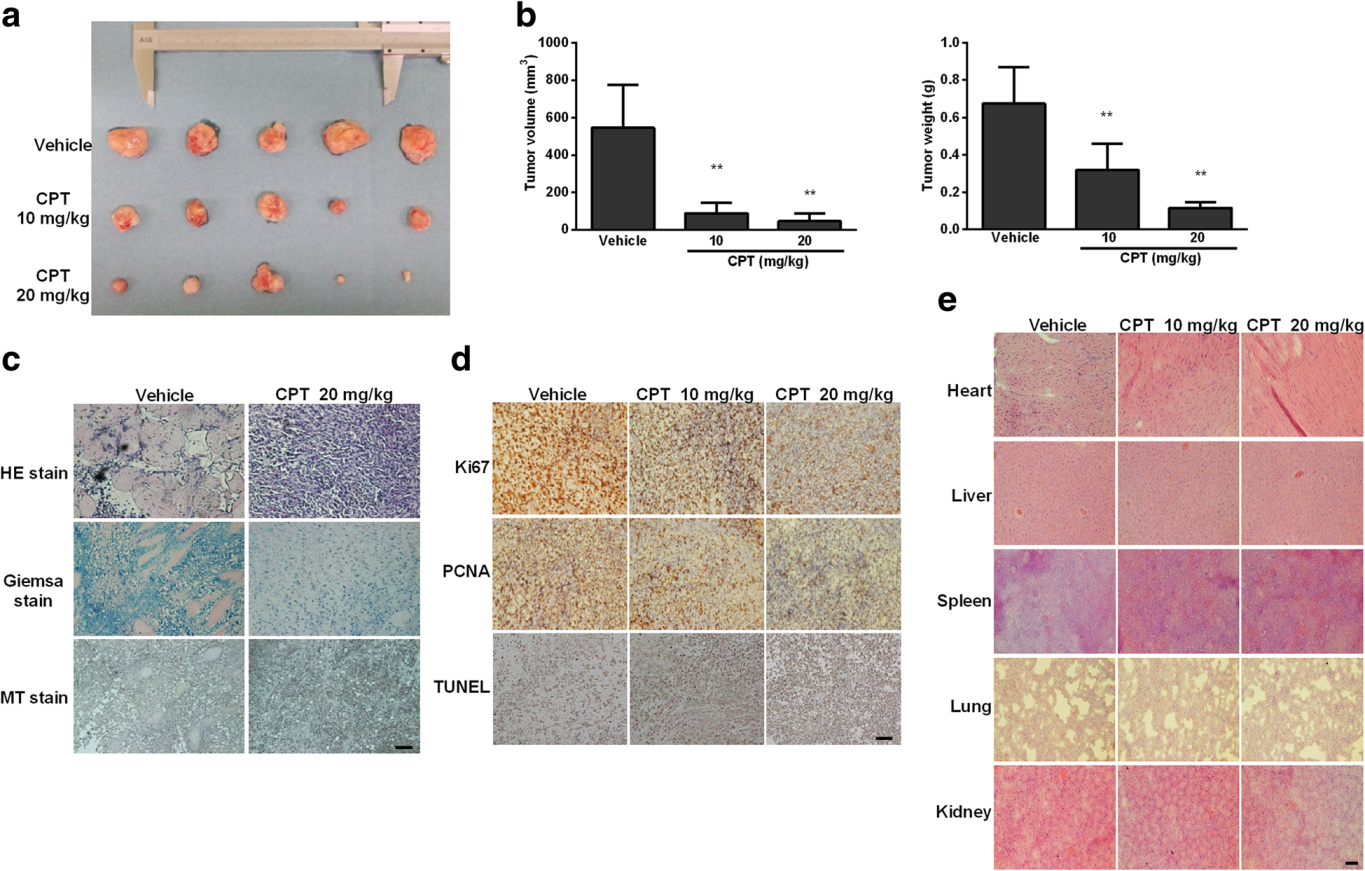
In vivo evidence for CPT inhibits OS growth. **a-b** 143B cell-derived tumors were developed in nude mice and treated with vehicle or CPT. Tumor growth was monitored by measuring the tumor volume and tumor weight for 45 days (*n* = 5 mice/group; **P < 0.01). Representative tumor images are shown here. **c** Tumors dissected from vehicle and CPT-treated mice were subjected to H&E, Giemsa and Masson’s trichrome (MT) stains. **d**Tumors were subjected to immunohistochemical analysis using Ki-67 and PCNA antibodies. Determination of apoptosis in tumor tissues by TUNEL assay. **e** Administration of CPT exhibited no toxicity in five major organs. H&E staining was used to evaluate the histology. Bar represents 500 μm

In order to investigate the effects of CPT on tumor growth in vivo, NOD-SCID mice were treated with or without IP injection of CPT (10 mg/kg or 20 mg/kg) every other day for a total of 45 days. As shown in Fig. 2a and b, CPT-treated tumor tissues showed significant decreases in volume and weight. To examine the changes of tumor cell morphology between the control and CPT-treated groups, hematoxylin and eosin (H & E) staining, Giemsa stain, and Masson’s trichrome stain were performed. The significant proliferation of osteoid with a high density of malignant cells was observed in the vehicle control group, but not in the CPT-treated group (Fig. 2c). Immunohistochemistry staining of PCNA and Ki67, and TUNEL staining were used to detect cell proliferation and apoptosis, respectively. We found the levels of both PCNA and Ki67 were notably decreased, whereas the level of TUNEL-positive cells was increased (Fig. 2d). To investigate any potential cytotoxicity of CPT on normal tissues, tumor-bearing mice were intraperitoneally treated with CPT, and H&E staining of organs were included at the end of the experiment, revealing no specific organ-related toxicities (Fig. 2e). These data clearly demonstrate that CPT exhibits potent antitumor activity with insignificant toxicity in vivo*.*

### CPT induced mitochondrial fragmentation in OS cells

**Fig. 3:**
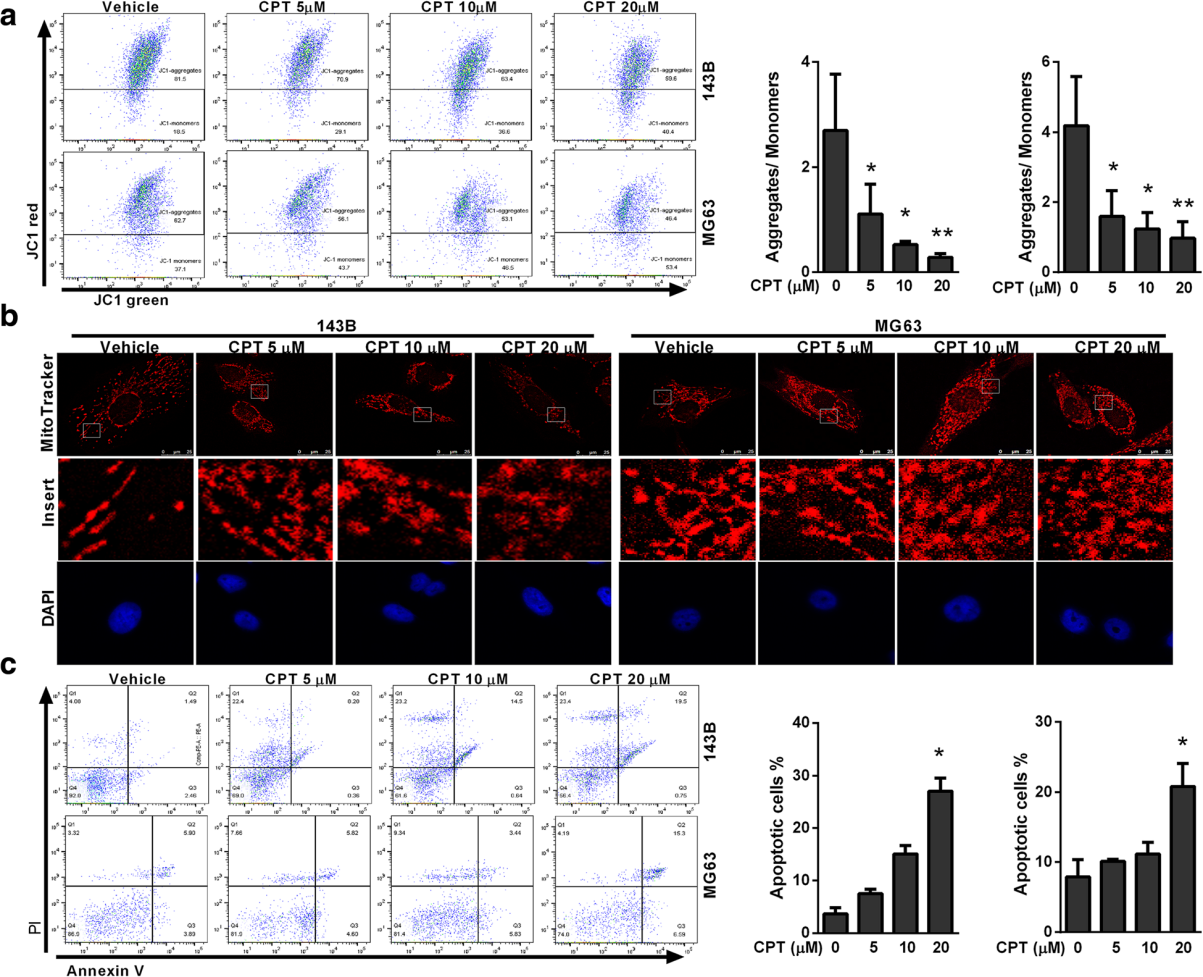
CPT triggers mitochondrial fragmentation and apoptosis in OS cells. **a** Representative dot plots of JC-1 aggregates versus JC-1 monomers in response to various CPT treatments of OS cells. **b** Representative changes in mitochondrial morphology were detected by confocal microscopy at various concentrations of CPT (as indicated). Images of mitochondria (red) and nucleus (blue) were collected by confocal microscope. **c** Annexin V/PI analysis by flow cytometry followed by CPT treatment (as indicated). The respective cell percentages in early and late apoptosis for different dose treatment are presented in the qantitative analysis. The results were expressed as the means ± SD from three independent experiments. *P < 0.05 and **P < 0.01, significantly different compared with control

The Δψm is a key indicator of cell health, or can precede externalization of phosphatidylserine and coincide with caspase activation under special circumstances. To examine the transition in mitochondrial membrane potential (MMP), a hallmark of apoptosis, osteosarcoma cells were stained with the fluorescent dye JC-1. After CPT treatment, JC-1 existed more in monomeric form and stained the cytosol green, which indicated decreased MMP in response to CPT (Fig. 3a). The mitochondria in these CPT-treated cells were stained by mitochondrion-specific dye MitoTracker Red. Our results showed that MitoTracker Red produced punctate mitochondrial staining, which indicated CPT gradually converted mitochondria into punctate morphology in a dose-dependent manner (Fig. 3b). Annexin V/PI staining further showed that the apoptosis rates in CPT-treated OS cells were significantly higher than those of control cells (Fig. 3c), indicating that CPT activated the process of apoptosis. These results clearly indicate that CPT effectively induces cell death, accompanied by mitochondrial demise, in human osteosarcoma cells.

### CPT induced activation of apoptotic pathway in osteosarcoma cells

**Fig. 4:**
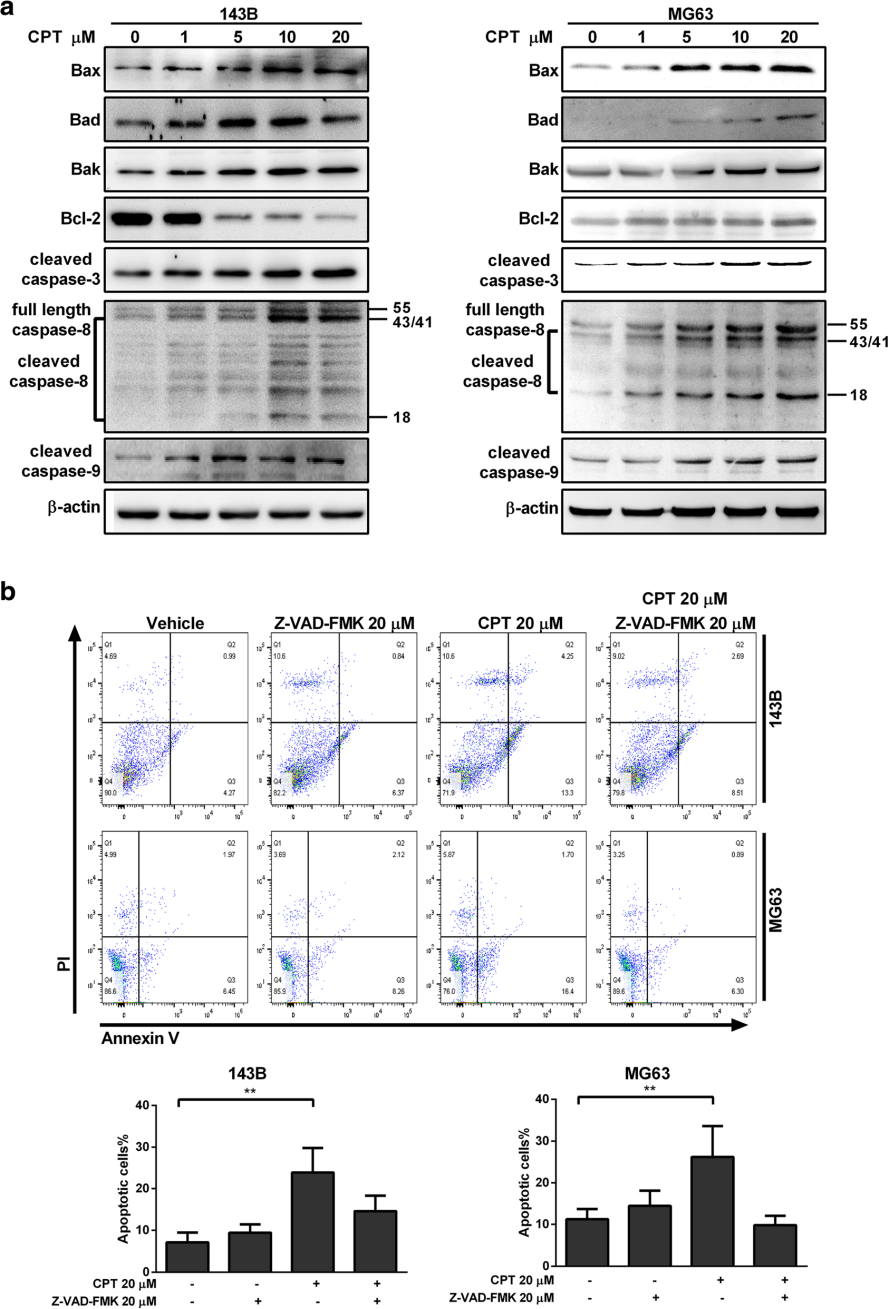
Caspase-3, − 8, and − 9 are involvement in CPT-induced OS cell apoptosis. **a** The expressions of apoptosis-related proteins were measured by western blotting in OS cells following 20 μM CPT treatment for 24 h. **b** OS cells were pretreated with Z-VAD-FMK (20 μM) for 1 h and incubated with 20 μM CPT for 24 h. Annexin V/PI analysis was used to detect apoptotic cells by flow cytometry followed by CPT treatment. The respective cell percentages in early and late apoptosis for different dose treatment are presented in the quantitative analysis. The results were expressed as the means ± SD from three independent experiments. **P < 0.01, significantly different compared with control

The intrinsic apoptosis (mitochondrial) pathway can be initiated through caspase-9, while the extrinsic pathway is mediated by caspase-8. We therefore explored the effects of CPT on the apoptotic pathway and cell death in osteosarcoma cells. As shown in Fig. 4a, notable increases in the activation of cleavage caspase-3, − 8, and − 9 were detected in immunoblot analysis of the CPT-treated OS cells compared to the control group. In line with this, co-treatment of 20 μM general caspase inhibitor Z-VAD-FMK abolished the ability of CPT to induce cell death, suggesting a caspase-dependent cell death following CPT treatment. Next, we investigated the apoptotic members of the Bcl-2 family, Bcl-2, Bax, Bad, and Bak, which are essential for many pathways associated with programmed cell death. These results demonstrated that CPT treatment of OS cells for 24 h led to the up-regulation of pro-apoptotic Bax, Bad, and Bak expressions, while anti-apoptotic Bcl-2 was down-regulated in a dose-dependent manner in 143B but not MG63 cells (Fig. 4a). Instead, CPT decreased Bcl-2 expression in MG63 cells at 36 h after treatment (Additional file 4: Figure S3). Thus, these data indicate that CPT triggers cell death by activating the intrinsic pathway.

### Drp1 is required for Bax-mediated intrinsic apoptosis

**Fig. 5:**
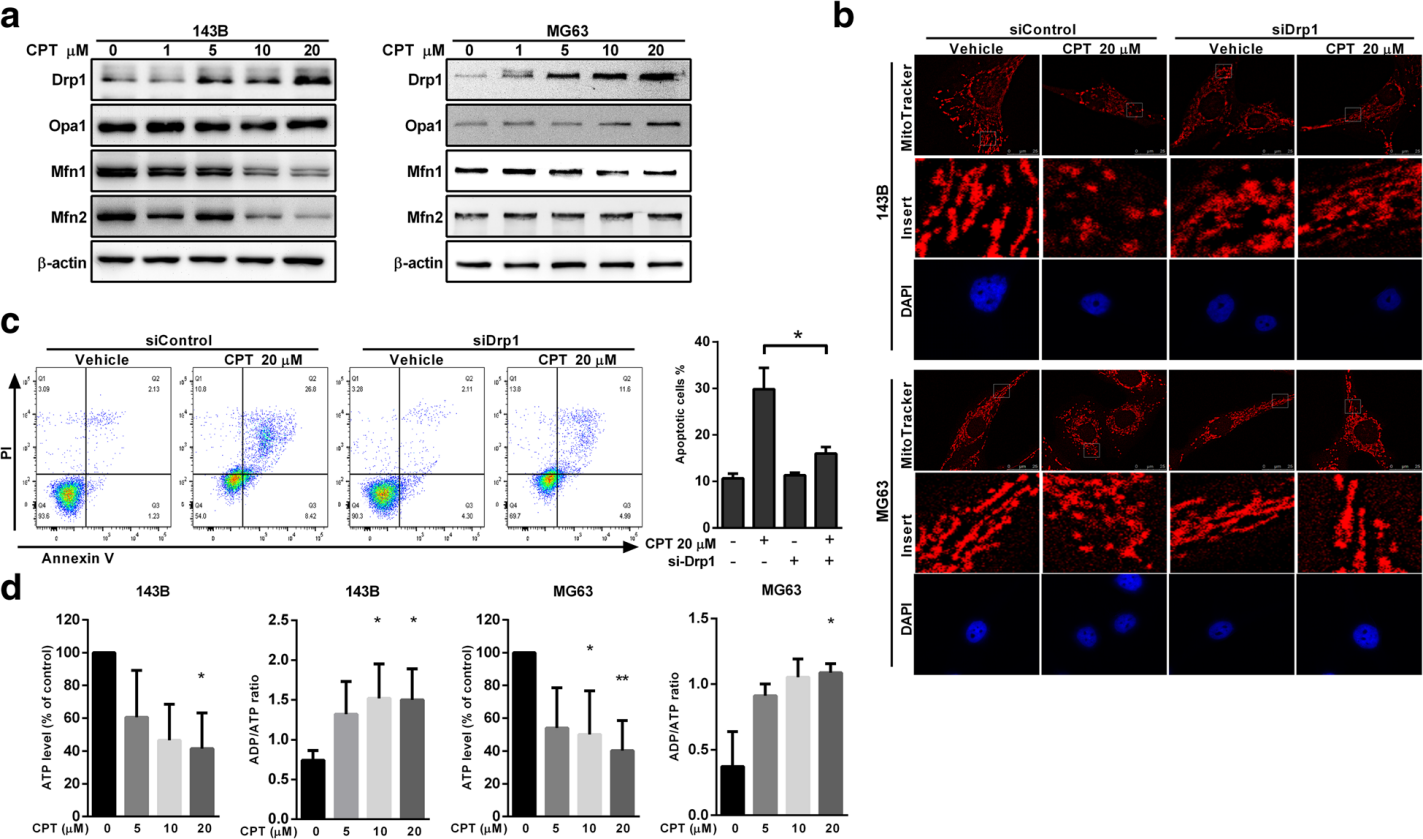
Drp1 silencing attenuates CPT-induced mitochondrial fragmentation and apoptosis. **a** The effects of CPT on mitochondrial fusion/fission proteins expression in OS cells. Representative immunoblot of the protein levels of Drp1, Opa1, Mfn1 and Mfn2. **b** Comparison of mitochondrial morphology in the control and Drp1 siRNA cells. Images of mitochondria (red) and nucleus (blue) were collected by confocal microscope. **c** Drp1 silencing reversed the reduction of cell viability in CPT treated 143B cells. Representative dot plots of Annexin V/PI analysis after CPT treatments in the presence or absence of Drp1 siRNA. **d** CPT decreased ATP production and increased ADP/ATP ratio of both 143B and MG63 cells. The results were expressed as the means ± SD from three independent experiments. *P < 0.05, significantly different compared with control

Mitochondrial dynamics are dysfunctional in many diseases including cancer, and influencing/affecting this dysfunction has recently been proposed as a therapeutic strategy for the treatment of tumors. In addition, new evidence has emerged that mitochondrial fission plays a corroborative component in apoptotic cell death [26]. In the present study, we further investigated the effects of CPT on the expression of mitochondrial fission protein (Drp1) and fusion proteins (Mfn1, Mfn2 and Opa1) in OS cells. We observed an upregulation of Drp1, but a decrease of Mfn1, Mfn2, and Opa1 following CPT treatment (Fig. 5a). Consequently, after endogenous knockdown of Drp1via Drp1-siRNA (Additional file 5: Figure S4), silencing of Drp1was found to efficiently reverse mitochondrial fragmentation in the cells treated by 20 μM CPT (Fig. 5b). At the same time, CPT-induced cell death of 143B cells was reversed, similar with the control group (Fig. 5c). In order to evaluate the energy production related to mitochondrial dysfunction, we further detected the ATP levels and ADP/ATP ratio of both 143B and MG63 cells. We found that CPT decreased ATP production as well as increasing ADP/ATP ratio of both OS cells (Fig. 5d). These results indicate that CPT-induced Drp1 upregulation results in mitochondrial fission, which contributes to OS cell death.

### CPT induced a time-associated increase in the interaction between active Bax and Drp1

**Fig. 6:**
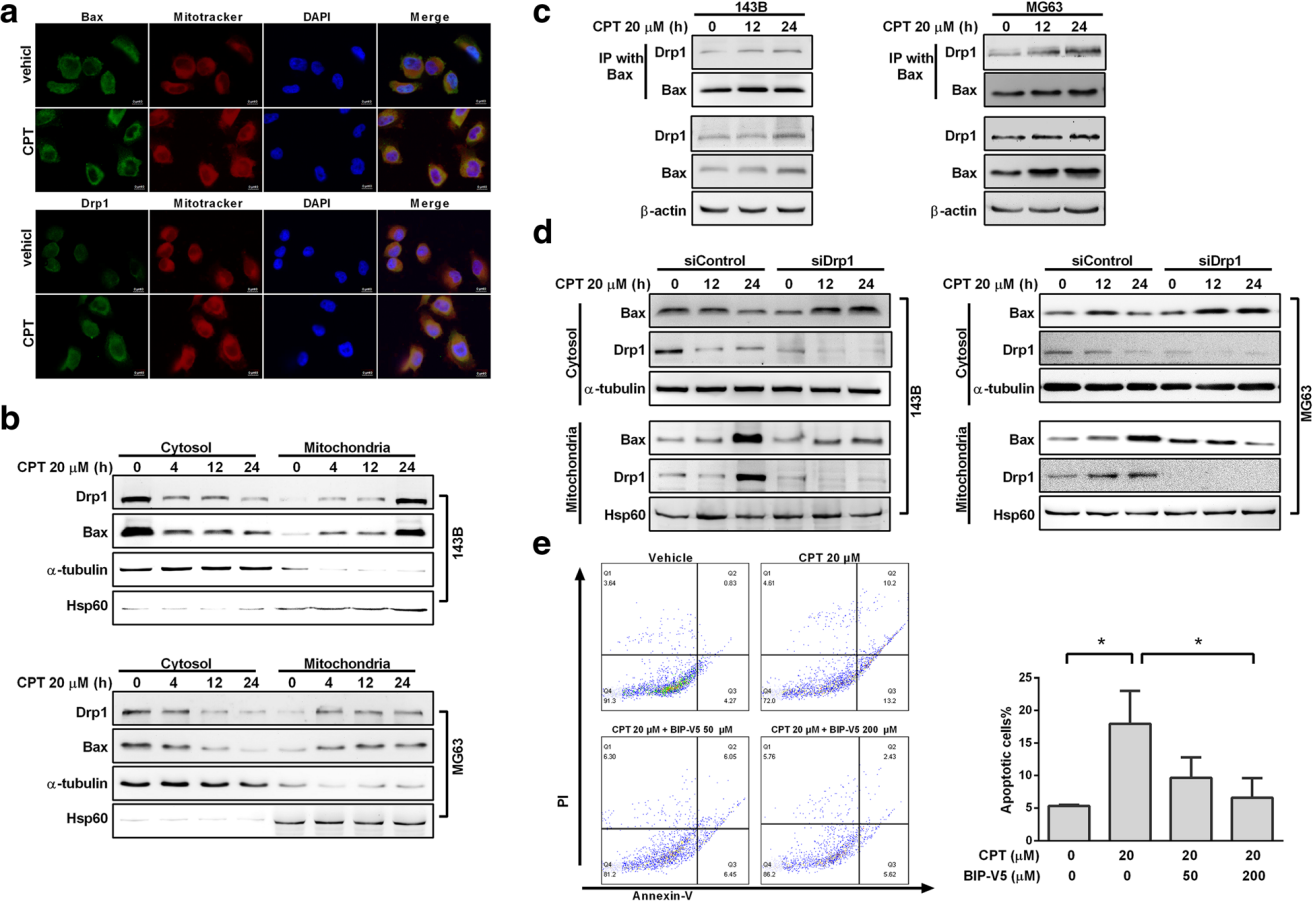
Drp1 is required for CPT-mediated apoptosis. **a** Immuno-staining of Bax and Drp1. Confocal microscope scanning photographs indicated the mitochondrial localization of Drp1 and Bax in CPT treated 143B cells. Red: Mito-Tracker Red CMXRos, Green: Bax and Drp1 as indicated. **b** Expression of Drp1 and Bax in cytosolic and mitochondrial fractions in OS cells were determined by immunoblotting using anti-Drp1 and anti-Bax antibodies. **c** Interaction between Bax and Drp1 after CPT treatment. Proteins were extracted from CPT-treated OS cells, and then IP was performed with Bax antibody; Co-IP Drp1 and Bax was detected by Western blotting. **d** After knocking down Drp1 for 24 h, OS cells were treated with CPT 20 μM for indicated time. Bax expression was determined in both cytosolic and mitochondrial fractions using mouse anti-Bax antibody. Hsp60 and α-tubulin antibodies were used as loading controls for mitochondria and cytosol, respectively. **e** 143B cells were pretreated with Bax inhibitor peptide V5 (BIP-V5) (50 or 200 μM) for 1 h and incubated with 20 μM CPT for 24 h. Annexin V/PI analysis was used to detect apoptotic cells by flow cytometry followed by CPT treatment. The respective cell percentages in early and late apoptosis for different dose treatment are presented in the qantitative analysis. The results were expressed as the means ± SD from three independent experiments. *P < 0.05, significantly different compared with control

The present study indicated that Drp1 is recognized as a critical protein leading to mitochondrial fragmentation which facilitates Bax insertion and eventual cell death. In our previous result, shown in Fig. 5, we found that CPT-triggered mitochondrial fragmentation, which preceded mitochondrial dysfunction. Consistent with excessive mitochondrial fragmentation, CPT exposure concomitantly increased the protein levels of Drp1 and Bax, thus justifying further investigation of the effects and underlying mechanisms of CPT-induced Drp1 on mitochondrial dynamics. Confocal microscopy and imaging colocalization analysis showed that CPT exposure promoted both Drp1 and Bax translocation from the cytoplasm into mitochondria in 143B cells (Fig. 6a). To further analyze the protein dynamics of Drp1 and Bax in cytoplasm and mitochondria, differential detergent fractionation technique (DDF) was used. As shown in Fig. 6b, we found significantly increased expression levels of Drp1 and Bax in the mitochondrial fraction, but decreased expressions in the cytosolic fraction of 143B and MG63 cells. Next, to determine whether CPT could trigger Bax and Drp1 to bind directly, co-IP was performed in 143B and MG63 cells. The immunoprecipitated Drp1 increased time-dependently as baited/in conjunction with Bax under CPT treatment (Fig. 6c). To determine whether Drp1 is required for Bax translocation to mitochondria in response to CPT-induced apoptosis, expression of Drp1 was partially knocked down by transfection with siRNA-Drp1. In response to CPT treatment, Bax levels were decreased in the cytosolic fraction, and were increased in the mitochondrial fraction of the OS cells transfected with control siRNA, indicating that Bax mitochondrial translocation occurred (Fig. 6d). Importantly, Bax in the Drp1-deficient cells was accumulated in the cytosolic fraction, and the levels of mitochondrial Bax remained relatively unchanged compared to the control group, suggesting that Bax translocation to mitochondria was blocked. Considering that the Drp1-Bax axis is essential to the anticancer effect of CPT, we investigated whether BIP-V5 (Bax inhibitor) could abrogate this effect. As shown in Fig. 6e, CPT treatment led to the death of OS cells, and the blockage/impeding of Bax could impair CPT-induced apoptosis. Taken together, these data suggest that CPT enhances the interaction between Drp1 and Bax, which is responsible for CPT-induced apoptosis in osteosarcoma cells.

### CPT is a potent inducer of intrinsic apoptotic pathway and mitochondrial fission in vivo

**Fig. 7:**
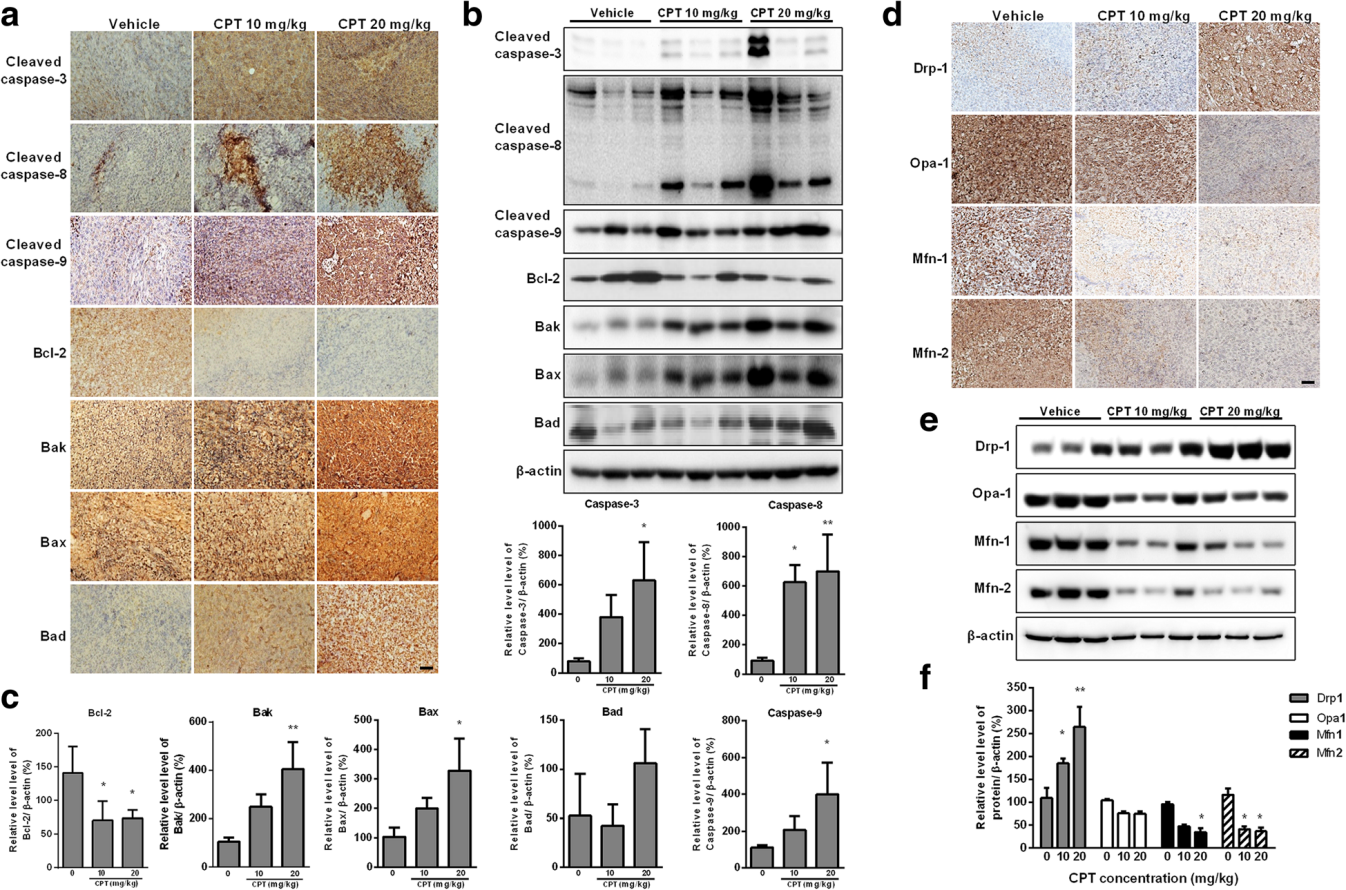
CPT activates caspase-mediated apoptosis and impaired balance of mitochondrial fission and fusion in vivo. 143B cell-derived tumors were developed in NOD/SCID mice and treated with CPT or vehicle for 45 days. Expressions of cleaved caspase 3, cleaved caspase 8, cleaved caspase 9, Bcl-2, Bak, Bax and Bad were examined by immunohistochemistry (**a**) and Western blotting (**b**). The quantification analysis in Figs. **b** is for Figs. (**c**). Expressions of Drp1, Opa1, Mfn1 and Mfn2 were examined by immunohistochemistry (**d**) and Western blotting (**e**). The quantification analysis in Figs. **e** is for Figs. (**f**). Ratios of each protein to β-actin were determined by densitometry. The results were expressed as the means ± SD from three independent experiments. *P < 0.05 and **P < 0.01, significantly different compared with control. Bar represents 500 μm.

**Fig. 8:**
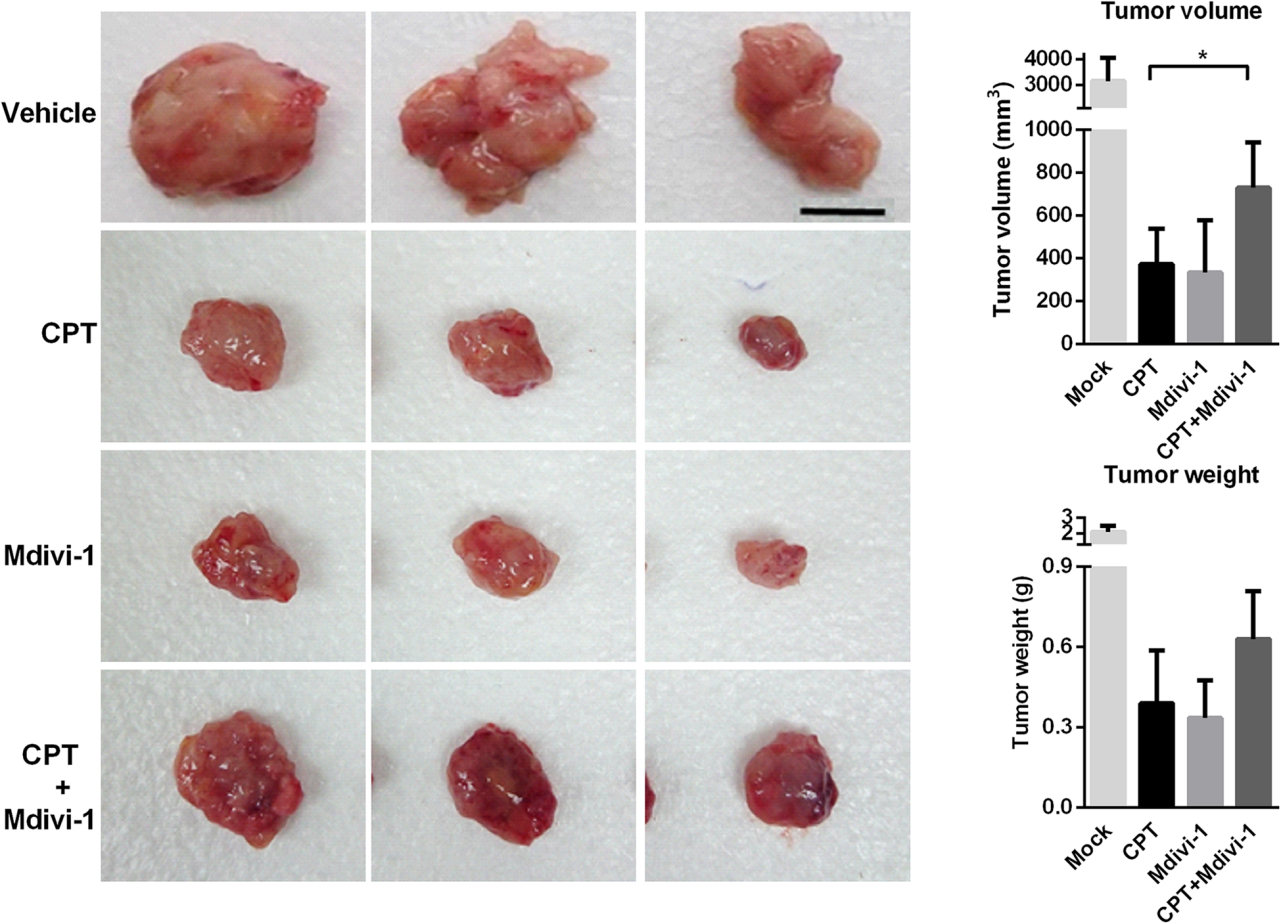
Antitumor ability of CPT was Drp1 dependent. Efficacy of CPT in combination with Mdivi-1 in xenograft model bearing 143B cells. 143B cells were inoculated subcutaneously in NOD/SCID mice, tumors were allowed to form, and then mice were treated with CPT combined with or without Mdivi-1 (0.5 mg/mice). Representative tumor images are shown here. Tumor volumes and tumor weight were examined throughout the experiment. (*n* = 3 mice/group; *P < 0.05). Bar represents 1 cm

To evaluate the antitumor effect of CPT in vivo, a xenograft OS model was generated. Tumors from CPT-treated mice exhibited increased cleaved cappase-3, cleaved caspase-8, and cleaved caspase-9 as well as higher expressions of Bak, Bax and Bak; whereas the level of Bcl-2 was decreased in comparison with the vehicle control group (Fig. 7a). In further investigation, Western blot experiments were conducted to complement the results of IHC (Fig. 7b and c), which found results consistent with those obtained by IHC. Furthermore, the significantly increased protein levels of Drp1, but decreased mitochondrial fusion proteins Mfn1, Mfn2, and Opa1, were observed in a dose-dependent manner in CPT-treated mice (Fig. 7d, e and f). Mdivi-1, a selective cell-permeable inhibitor of mitochondrial division Drp1, was used in the xenograft NOD/SCID mice model. Tumor growth was significantly inhibited in the CPT or Mdivi-1 alone-treated group, whereas Mdivi-1 restored the tumor growth in the CPT and Mdivi-1combined treatment group (Fig. 8). Mdivi-1treatment mitigates CPT-induced antitumor activity. These findings are consistent with our in vitro studies showing that CPT induced cancer cell death is Drp1 dependent. Altogether, CPT exhibits potent antitumor activity in association with induction of caspase-dependent apoptotic pathways, and alters the balances of mitochondrial fission and fusion.

## Discussion

Investigations into mitochondrial biology and tumorigenic signaling are revealing novel approaches to developing targeted cancer therapy. The present study provides evidence that CPT exposure led to pronounced mitochondrial fragmentation in 143B and MG63 osteosarcoma cells. Additionally, CPT-triggered mitochondrial fragmentation preceded mitochondrial damage in vitro and was associated with CPT-induced cancer cell apoptosis in vivo. We report for the first time that CPT promotes Drp1 interaction with Bax, triggering Bax translocation into mitochondria, which is crucial for CPT-triggered cancer cell death. Autophagy-related protein associated with antitumor properties of CPT, in particular, needs further investigation (Additional file 6: Figure S5).

The core components of the fusion and fission machineries/mechanisms have been identified as the dynamin-related GTPases Drp1, mitofusins (Mfn) 1 and 2, and Opa1 [27]. Multiple studies have demonstrated that enhanced fission or reduced fusion, high expression or enhanced activation of Drp1, and/or downregulation of MFN2 are linked to several cancer-related phenotypes [28, 29], indicating that cancer cells often exhibit fragmented mitochondria [30]. However, Drp1^−/−^ embryos showed considerably weaker signals and decreased numbers of caspase-3–positive cells, demonstrating the physiological role of Drp1 as facilitating developmentally-regulated apoptosis during neural tube formation [31]. The role of Drp1 in tumorigenesis thus may appear to be paradoxical, since mitochondrial fission plays a key mediator of two very distinct processes, cellular apoptosis and cell mitosis [32]. It has been demonstrated that mitochondrial dynamics (both fission and fusion) act as a rheostat that determines apoptotic susceptibility, as loss of Drp1 postpones cytochrome c release and apoptotic induction, while a follow-up study indicated that fission was not required for Bax/Bak-mediated apoptosis [33]. Instead, a GTPase-independent function of Drp1 in membrane remodeling and hemi-fusion results in Bax oligomerization and subsequent MOMP, indicating that the death function of Drp1 can promote apoptosis independent of fission [34]. The role of Drp1 has been detected in complexes with Bax at mitochondria. In response to many apoptotic stimuli, activation of the pro-apoptotic Bax results from a highly regulated multistep process involving its translocation from the cytosol to the OMM, where it integrates and oligomerizes [35]. Although the exact mechanism by which Bax actively moves from the cytosol to the mitochondria is still unclear, recent studies have suggested that irradiation could induce a time-associated increase in the interaction between active Bax and Drp1, then Bax-Drp1complex translocate to discrete foci on the mitochondria, where mitochondrial Bax stabilizes Drp1 [11, 36, 37]. Our results support that CPT acted as an effective Drp1 activator, capable of inducing cancer cell death via direct interaction with Bax to participate in apoptotic fragmentation of mitochondria. Furthermore, 143B cells was more sensitive towards the toxicity of CPT than MG63 cells (Fig. 1). Our results indicated that Bcl-2 was decreased in a dose dependent manner after 24 h exposure of CPT in 143B but not MG63 cells. However, a declining trend of Bcl-2 expression in MG63 cells was observed following long-term exposure (36 h) of CPT (Additional file 4: Figure S3C). This discrepancy may be due to the cell-specific expression of Bcl-2.

Aside from apoptosis, the cell cycle is documented to alter mitochondrial dynamics. Mitochondrial fission increasingly occurs during cellular division, thus assuring equal segregation of mitochondrial contents in daughter cells. Drp1 has been recognized to be functionally or molecularly linked to Cyclin B, E, and D [38–40]. As a previous study has suggested, during mitosis, CDK1/cyclin B phosphorylates Drp1 at Ser^616^ to induce mitochondrial fission and proper organelle segregation [41]. On the other hand, mitochondria morphology was found to regulate the cell cycle, as the genetic inhibition of Drp1 and the use of Drp-1 inhibitor (mitochondrial division inhibitor 1, Mdivi-1) have led to a decrease in the number of cells in S phase and an increase in the number of cells in G2 phase [28]. The G2/M cell cycle arrest and aneuploidy were also observed in Drp1-deficient cells [42]. Crosstalk between the mitochondrial fission protein, Drp1, and the cell cycle is identified to play a critical role in the regulation of cell cycle progression. In response to CPT treatment, the present study found that osteosarcoma cells accumulated in S phase and significantly increased apoptosis rates, which could be rescued by knockdown Drp1 expression. Our results provide evidence that CPT-induced Drp1 expression is correlated with CPT-mediated S phase arrest and apoptosis induction.

Translocation, foci formation, and Bax activation, on the OMM leads to permeabilization and causes the release of proapoptotic factors from the mitochondrial intermembrane space to the cytosol [43]. Interestingly, not only does Bax promote the foci formation of Drp1, it also forms apoptotic mitochondrial localized foci that colocalized with Mfn1 and Mfn2 [44], preventing further fusion [36]. We herein found increased Drp1 in response to CPT treatment in both 143B and MG63 cells, while the expressions of Mfn1 and Mfn2 were decreased in 143B but not MG63 cells. We suppose the discrepancy may be a result of the differing proliferation rates of osteosarcoma. Drp1 expression patterns associated with cancer have been documented in several tumor models [32, 45, 46]. Tanwar et al. claimed that Drp1-based-gene-expression-signature could be used to recognize patients with poor survival possibilities from the primary tumors [47]. Also, Drp1 is essential in Ras-driven tumor growth and poor survival rate is associated with an increased Drp1 level reveal that Drp1 interacts with different biological processes in the tumorigenesis context [48]. Inhibition of cancer cell growth and/or enhanced spontaneous apoptosis induced by Drp1 inhibition have been observed both in vitro and in vivo in several cancer types [28, 42, 49]. The researchers found that treatment with Mdivi-1 resulted in mitochondrial hyper fusion and chronic elevation of cyclin E, which prevented the progression of the cell cycle in human colorectal carcinoma cell line [50]. In agreement with this result, cell apoptosis induced by Mdivi-1 has been reported in human ovarian, breast cancer cell lines and xenograft models of lung cancer [28, 51, 52]. Consistent with this notion, we did find Mdivi-1 treatment caused a marked reduction of xenograft tumors developed from 143B cells (Fig. 8). These results suggest that Drp1 contributes to initial tumor growth rather than later phase of tumor progression. Intriguingly, investigations have revealed that abnormal mitochondrial fission, mediated by Drp1, leads to excessive mitochondrial fragmentation, which appears to be a requisite step in intrinsic apoptosis pathways [26, 53]. A possible explanation might be that upregulated mitochondrial fission may function as an important point of convergence in mediating oncogenic signaling and promoting cancer cell growth. We supposed that CPT induced Drp1 activation caused the imbalance of fission and fusion impacting mitochondrial function which attributes to apoptotic signaling of cancer cell death. As enhanced mitochondrial fission and impaired fusion appear to contribute fundamentally to the inhibition of certain cancers, Drp1-mediated mitochondrial fission thus may represent a promising novel therapeutic target for cancers demonstrating excessive mitochondrial fission.

## Conclusions

In summary, our study provides further evidence that CPT triggers Drp1 expression to activate mitochondrial fission, which results in Bax activation and downstream intrinsic apoptosis, effectively inhibiting osteosarcoma growth. Therefore, investigation into CPT-induced inhibition of osteosarcoma cell growth suggests that influencing mitochondrial fission/fusion machinery may offer a novel approach to the development of future therapeutic cancer treatments.

## Additional files

Additional file 1: **Table S1.** The siRNA sequences of Drp1. (DOCX 15 kb)

Additional file 2: **Figure S1.** Cell viability of normal cells including mouse mesenchymal stem cell (MMSC), H184 and HaCaT cells following treatment with the various concentrations of CPT for 24 and 48 h. CCK-8 assay was used to assess cells proliferation. The results were expressed as the means ± SD from three independent experiments. (TIF 102 kb)

Additional file 3: **Figure S2.** The expression of cyclin-like proteins were measured by western blotting in OS cells treated with indicated concentration of CPT for 18 h. (TIF 799 kb)

Additional file 4: **Figure S3.** (A and B) The protein expressions were quantified as the expression ratio vs β-actin (Data represents the means ± SD from three independent experiments. *P < 0.05 and **P < 0.01, significantly different compared with control). (C) The protein expression of Bcl-2 was measured by western blotting in MG63 cells following CPT treatment for 36 h. (TIF 517 kb)

Additional file 5: **Figure S4.** siDrp1 was transfected into 143B cells and the transfectants were identified. β-actin served as loading control. (TIF 132 kb)

Additional file 6: **Figure S5.** Induction of autophagy in human osteosarcoma cells following CPT treatment. (A) Conversions of LC3B-I to LC3B-II were determined by immunoblotting following treatment with various concentrations of CPT in OS cells for 24 h. β-actin served as loading control. (B) Effect of silencing LC3 on CPT-mediated 143B cell growth. si-LC3 RNA was transfected into 143B cells and the transfectants were identified. CCK-8 assay was used to assess 143B cell proliferation. The results were expressed as the means ± SD from three independent experiments. *P < 0.05, significantly different compared with control. (TIF 369 kb)

## Abbreviations

CCK-8: Cell counting kit-8
CPT: Cryptotanshinone
DAPI: 4′,6-diamidino-2-phenylindole dihydrochloride
Drp1: Dynamin-related protein 1
H&E: Hematoxylin and eosin stain
JC-1: 5,5′, 6,6′-tetrachloro-l,1′,3,3′-tetraethylbenzimidazolylcarbocyanine iodide
Mfn1 and 2: Mitofusin1 and 2
MOMP: Mitochondrial outer membrane permeabilization
MT stain: Masson’s trichrome stain
OMM: Outer mitochondrial membrane
Opa1: Optic atrophy1
OS: Osteosarcoma
siRNA: Small interfering RNA
TUNEL: Terminal deoxynucleotidyl transferase dUTP nick end labeling
ΔΨm: Mitochondrial membrane potential
