# Supplementary material for: Activation of dynamin-related protein 1 - dependent mitochondria fragmentation and suppression of osteosarcoma by cryptotanshinone
Source: J Exp Clin Cancer Res. 2019 Jan 28;38:42. doi: 10.1186/s13046-018-1008-8 (PMC6350405; doi:10.1186/s13046-018-1008-8)
Supplement: Supplementary file 1 — Table S1. The siRNA sequences of Drp1. (DOCX 15 kb) [file 13046_2018_1008_MOESM1_ESM.docx]

| **Gene specific ChimeraRNAi** | **sense** | **antisense** |
| --- | --- | --- |
| DNM1L | 5'-CCG UGA UGA GUA U(g ctt ttc t) -3' | 5'-(aa aag c)A UAC UCA UCA CGG AU -3' |

Supplementary Table 1. The siRNA sequences of DRP1.
