# Supplementary figures and images for: Activation of dynamin-related protein 1 - dependent mitochondria fragmentation and suppression of osteosarcoma by cryptotanshinone
Source: J Exp Clin Cancer Res. 2019 Jan 28;38:42. doi: 10.1186/s13046-018-1008-8 (PMC6350405; doi:10.1186/s13046-018-1008-8)

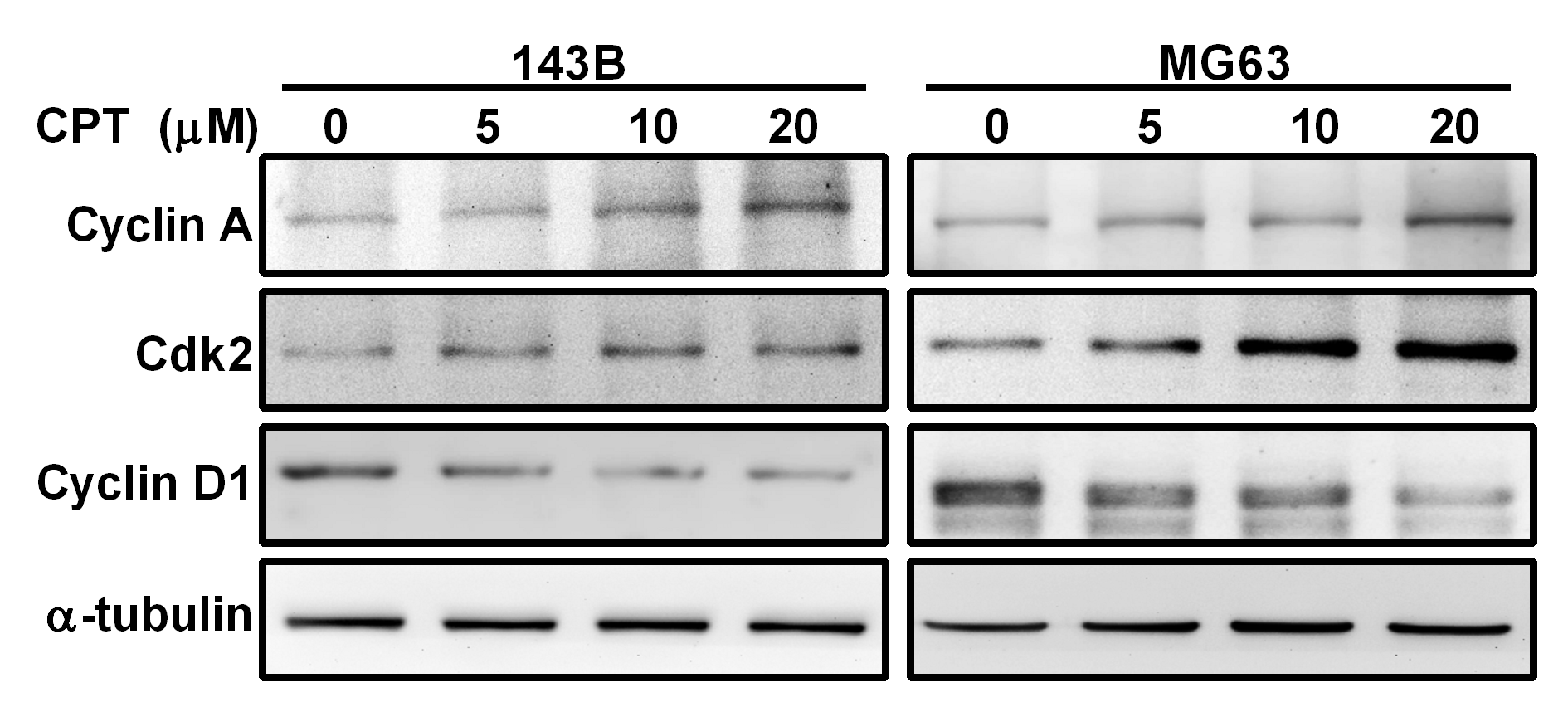

Supplement: Supplementary file 3 — Figure S2. The expression of cyclin-like proteins were measured by western blotting in OS cells treated with indicated concentration of CPT for 18 h. (TIF 799 kb) [file 13046_2018_1008_MOESM3_ESM.tif]

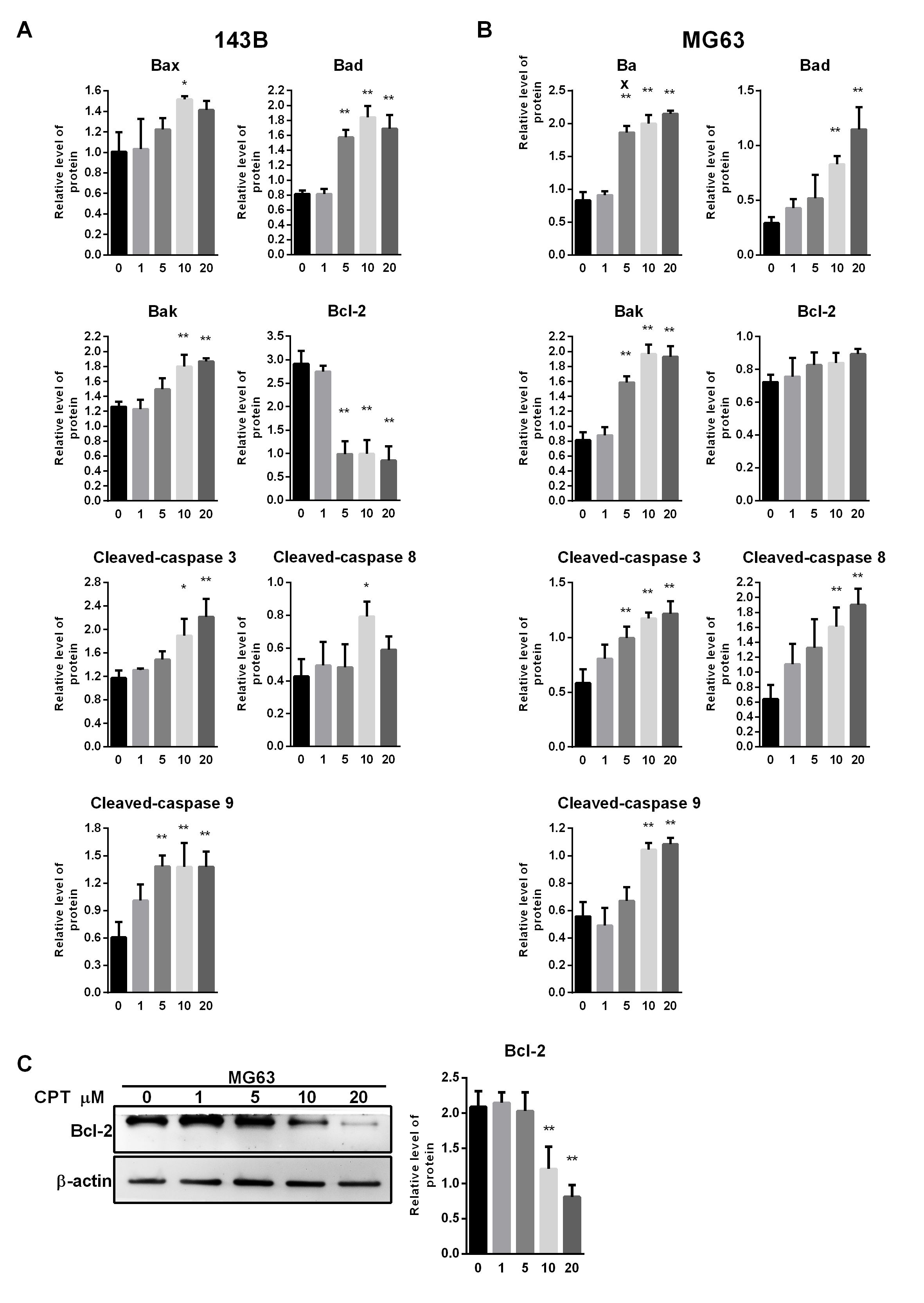

Supplement: Supplementary file 4 — Figure S3. (A and B) The protein expressions were quantified as the expression ratio vs β-actin (Data represents the means ± SD from three independent experiments. *P < 0.05 and **P < 0.01, significantly different compared with control). (C) The protein expression of Bcl-2 was measured by western blotting in MG63 cells following CPT treatment for 36 h. (TIF 517 kb) [file 13046_2018_1008_MOESM4_ESM.tif]

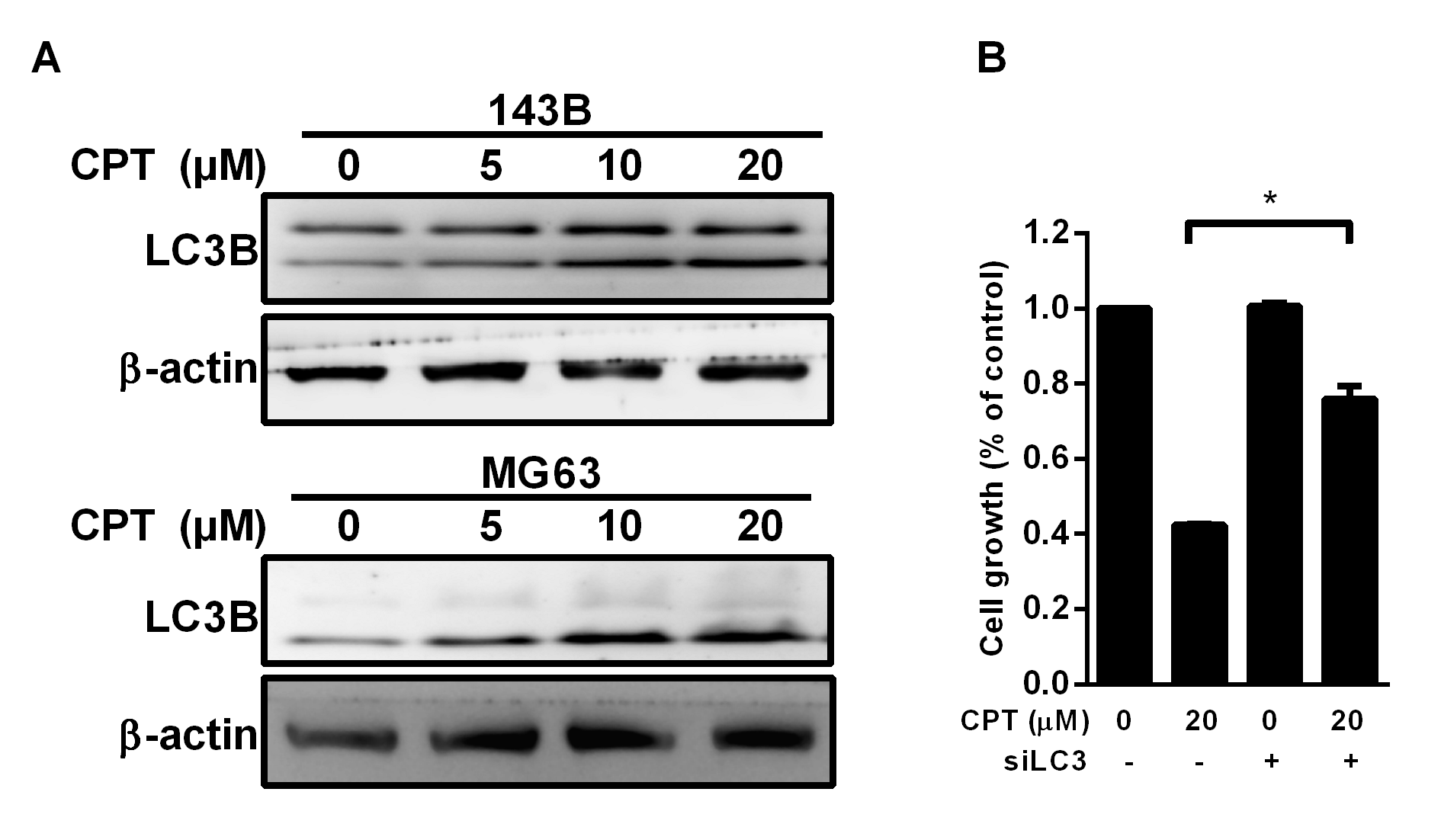

Supplement: Supplementary file 5 — Figure S4. siDrp1 was transfected into 143B cells and the transfectants were identified. β-actin served as loading control. (TIF 132 kb) [file 13046_2018_1008_MOESM5_ESM.tif]

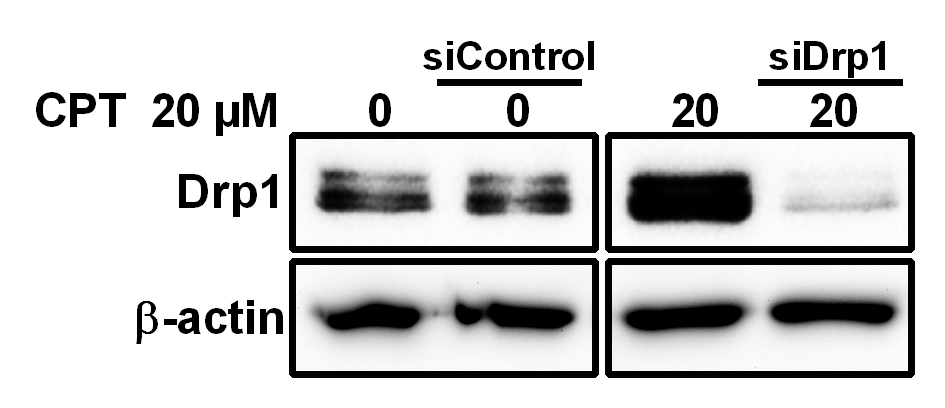

Supplement: Supplementary file 6 — Figure S5. Induction of autophagy in human osteosarcoma cells following CPT treatment. (A) Conversions of LC3B-I to LC3B-II were determined by immunoblotting following treatment with various concentrations of CPT in OS cells for 24 h. β-actin served as loading control. (B) Effect of silencing LC3 on CPT-mediated 143B cell growth. si-LC3 RNA was transfected into 143B cells and the transfectants were identified. CCK-8 assay was used to assess 143B cell proliferation. The results were expressed as the means ± SD from three independent experiments. *P < 0.05, significantly different compared with control. (TIF 369 kb) [file 13046_2018_1008_MOESM6_ESM.tif]
